# Clinical effect of decitabine in the treatment of myelodysplastic syndrome and influencing factors

**DOI:** 10.12669/pjms.36.5.2289

**Published:** 2020

**Authors:** Tangxia Liu, Jing Wang, Chunmei Li, Lingzhi Jia

**Affiliations:** 1 Tangxia Liu, Hematology Department, Binzhou People’s Hospital, Shandong, 256610, China; 2 Jing Wang, Mental Health Center, Binzhou People’s Hospital, Shandong, 256610, China; 3 Chunmei Li, Digestive Endoscopy Center, Binzhou People’s Hospital, Shandong, 256610, China; 4 Lingzhi Jia, Hematology Department, Binzhou Medical University Hospital, Shandong, 256603, China

**Keywords:** Decitabine, Myelodysplastic syndrome, CAG, Scheme

## Abstract

**Objective:**

To analyze the effect of decitabine combined with conventional chemotherapy on myelodysplastic syndrome (MDS) and its influencing factors.

**Methods:**

Eighty patients with MDS who were admitted to our hospital were selected by this study between February 2017 and February 2018. The selected patients were divided into a traditional group (CAG/DA scheme) and the combined group (DAC combined with CAG/DA scheme) according to the random number table method, 40 each. The clinical treatment effects of the two groups were compared, and the influencing factors of the effect were analyzed.

**Results:**

After four courses of treatment, the difference in the total effective rate between the two groups were statistically significant (P<0.05); the difference in the incidence of adverse reactions and in the overall survival (OS) rate between the two groups were not statistically significant (P>0.05). However, the progression free survival (PFS) rate of the combined group was significantly higher compared with the traditional group (P<0.05); the Hb level, WHO stage and and karyotype of the patients before treatment had significant influence on the treatment effect (P<0.05).

**Conclusion:**

DAC in combination with conventional chemotherapy has good effect in the treatment of MDS, and it will not increase adverse reactions. In addition to treatment scheme, the influencing factors of the effect of treatment for MDS also include Hb, WHO stage and karyotype.

## INTRODUCTION

Myelodysplastic syndrome (MDS) is a common disease in the blood system, which is mainly caused by the abnormal development of myeloid cells and ineffective hematopoiesis. It is characterized by the increase of primordial cells and the increase and morphological abnormality of of bone marrow nucleated cells.^1^,^2^ Clinically, there is a high risk of MDS turning into acute myeloid leukemia, and the incidence of MDS tends to increase with age.^3^ At present, there are many treatment methods for MDS, but there is no unified method. In recent years, a relevant research shows that DNA methylation is a common phenomenon in MDS patients and the inactivation of tumor suppressor gene caused by hypermethylation is closely related to the progress of MDS.^4^ The diagnosis and treatment guidelines of European Leukemia Net recommend methyltransferase inhibitors for treatment of MDS.^5^ Decitabine is a specific DNA methyltransferase inhibitor, which can induce tumor cells to turn into normal cells or to die.^6^,^7^ However, the clinical application time of decitabine in China is relatively short, and large sample data are not yet available to support whether factors such as Revised International Prognostic Scoring System (IPSS-R) risk stratification and genetic difference will affect the treatment effect of DAC for MDS. Therefore, this study explored the effect of DAC combined with conventional chemotherapy on MDS and its influencing factors, in order to provide more optimal choices for clinical practice.

## METHODS

Eighty MDS patients who were admitted to our hospital were selected as the study subjects from December 2017 to December 2018. All selected patients were divided into a traditional group (CAG/DA scheme) and a combined group (DAC combined CAG/DA scheme) according to the random number table method.

There were 40 patients in the traditional group, including 22 males and 19 females, and they aged 21-67 years, with an average age of (43.3±12.5) years. Before treatment, the level of peripheral white blood cells (WBC) was (17.5±5.3)×10^9^/L, the level of platelets (PLT) was (67.4±31.9)×10^9^/L, and the level of hemoglobin (Hb) was (72.3±18.9) g/L. As to the WHO stage, there were 24 cases of RAEB-1 stage and 16 cases of RAEB-2 stage. As to the IPSS score, there were 0 case of low risk, 26 cases of medium risk and 14 cases of high risk. As to the karyotype, there were 21 good cases, 13 moderate cases and six bad cases. There were 40 patients in the combined group, including 29 males and 11 females, and they aged 19-61 years, with an average age of (39.7±15.1) years. Before treatment, the level of WBC was (18.2±5.6)×10^9^/L, the level of PLT was (66.1±28.6)×10^9^/L, and the level of Hb was (70.8±16.2) g/L. As to the WHO stage, there were 22 cases of RAEB-1 stage and 18 cases of RAEB-2 stage. As to the IPSS score, there were 0 case of low risk, 23 cases of medium risk, and 17 cases of high risk. As to the karyotype, there were 18 good cases, 14 moderate cases and 8 bad cases. There was no significant difference in age, gender, WBC, Hb, PLT, WHO stage, IPSS score and karyotype between the two groups (P>0.05); therefore, the results were comparable. This study was approved by the ethics committee (Ref# 160, Dated: January 1, 2020) of our hospital.

### Inclusive criteria

(1) The diagnosis and classification of MDS patients referred to Expert Consensus on Diagnosis and Treatment of Myelodysplastic Syndrome of Chinese Society of Hematology of Chinese Medical Association and the WHO standard for hematolymph malignancies;^8^ (2) the patients were diagnosed after bone marrow puncture; (3) the patients signed the informed consent.

### Exclusive criteria

The exclusive criteria included: (1) transforming into leukemia; (2) having seriously damaged liver and kidney function which was difficult to treat; (3) the survival time shorter than 3 months; (5) failed follow-up observation; (4) having severe infection.

### Therapeutic method

F CAG/ DA scheme was used in the traditional group. CAG: aclacinomycin was intravenously injected, 20 mg/time, from the first day to the fifth day; cytarabine was intravenously injected at a dose of 15 mg/time, from the first day to the 14^th^ day, twice each day; recombinant human granulocyte colony-stimulating factor injection was intravenously injected at the dose of 300 μg/time, from the first day to the 14^th^ day, twice each day. DA: daunorubicin was intravenously injected at the dose of 60 mg/time, from the first day to the third day, twice each day; cytarabine was intravenously injected at the dose of 100 mg/time, from the first day to the fifth day, twice each day. The patients in the combined group were treated with the scheme of DAC combined with CAG/DA. The CAG/DA scheme was the same as above, and DAC was given 20 mg each time. Four courses were taken as one course, and the patients in the two groups were treated for four courses.

### Supportive treatment and nursing

The patients in the two groups were given symptomatic support treatment such as nutrition support, hydration, alkalization, vomit stopping and stomach care. For example, if Hb<60 g/L, suspension red blood cells were infused; if PLT<20×10^9^/L or there was bleeding tendency, platelet was infused; if granulocyte<0.5×10^9^/L or granulocytopenia was combined with severe infection, granulocyte colony-stimulating factor was infused; patients with infection was given anti-infection treatment. Moreover, comfort care intervention was also used. The specific contents included: (1) psychological nursing: once MDS patients were diagnosed, they were prone to produce negative emotions such as tension and anxiety; therefore the nursing staff patiently explained the basic knowledge, treatment measures, outcome and prognosis of the disease to the patients and their families and inform them of the possible adverse reactions, precautions and response measures caused by chemotherapy, so as to help the patients to stabilize their emotions; (2) diet nursing: patients were asked to eat foods rich in iron, protein and vitamins, have more meals a day and less food at each, and balance nutrition; (3) life nursing: patients were instructed to work and rest regularly, establish good living habits, exercise moderately, strengthen self-protection awareness, and prevent complications; (5) complication nursing during chemotherapy, nurses closely observed whether patients had anemia, bleeding, infection, etc.

### Evaluation of treatment effect

After the treatment, the clinical effect of the two groups was evaluated. According to IWG2006 standard which was revised according to the criteria of therapeutical effect of international working group, the treatment effect was divided into complete remission (CR), partial remission (PR), hematological improvement (HI) and no remission (NR). The total effective rate was calculated by: total effective rate=(CR+PR)/total number of cases×100%. The evaluation of adverse reactions referred to the CTCAE v3.0 standard adopted by the National Cancer Institute (NCI). The end point of the study was death or deadline of follow up. Progression free time (PFS) refers to the time from the beginning of treatment to disease progression or death. Overall survival (OS) refers to the time from the beginning of disease diagnosis to death or deadline of follow up.

### Statistical analysis

SPSS 23.0 was used. Mean ± standard deviation was used for statistical software. Paired-t test was used. The categorical data was compared using Chi-square test. Kaplan-Meier method was used for survival analysis. Difference was considered as statistically significant if P<0.05.

## RESULTS

The total effective rate of the combined group was 72.5%, which was significantly higher than that of the traditional group (40%) (P<0.05, Table I). There was no significant difference in adverse reactions between the two groups (P>0.05, Table II). The follow up ended on August 2019. The median follow-up time was 18 months (5-30 months). In the traditional group, the median OS was 18 (5-30) months, the median PFS was 8 (3-25) months, and 10 patients transformed into acute myeloid leukemia. In the combined group, the median OS was 19 (7-29) months, the median PFS was 11 (2-26) months, and five patients transformed into acute myeloid leukemia. There was no significant difference in OS between the two groups (X^2^=0.007, P>0.05). Compared with the traditional group, PFS in the combined group was significantly higher, and the difference was statistically significant (X^2^=6.862, P<0.05).

**Table-I:** Chemotherapy effect between two groups [n(%)].

Group	CR	PR	HI	NR	Total effective rate
Traditional group (n=40)	10(25.0)	6(15.0)	18(45.0)	6(15.0)	16(40.0)
Combined group (n=40)	19(47.5)	10(25.0)	8(20.0)	3(7.5)	29(72.5)
X^2^	/	/	/	/	5.971
P	/	/	/	/	<0.05

**Table-II:** Adverse reactions caused by chemotherapy between two groups [n(%)].

Group	WBC reduction	Hb reduction	Infection	Nausea and vomiting	Mucositis
Traditional group (n=40)	11(27.5)	8(20.0)	10(25.0)	19(47.5)	5(12.5)
Combined group (n=40)	14(35.0)	5(12.5)	8(20.0)	25(62.5)	10(25.0)
X^2^	0.544	0.437	0.549	1.283	1.227
P	>0.05	>0.05	>0.05	>0.05	>0.05

Among 80 cases of MDS, 45 cases were effective and 35 cases were ineffective. The results showed that the Hb level, WHO stage and chromosome karyotype of patients before treatment had a significant impact on the treatment effect of MDS, and the difference was statistically significant (P<0.05, Table III).

**Table-III:** QLQ-C30 scale score between two groups (point, Mean±SD).

Influence factor		Effective (n=45)	Ineffective (n=35)	X^2^	P
Age	≥60	11(24.4)	13(37.1)	0.749	>0.05
	<60	34(75.6)	22(62.9)		
Gender	male	31(68.9)	20(57.1)	0.407	>0.05
	female	14(31.1)	15(42.9)		
WBC	≥4 (×10^9^/ L)	34(75.6)	24(68.6)	0.281	>0.05
	<4 (×10^9^/ L)	11(24.4)	11(31.4)		
PLT	≥100 (×10^9^/ L)	14(31.1)	4(11.4)	3.802	>0.05
	<100 (×10^9^/ L)	31(68.9)	31(88.6)		
Hb	≥90 g/L	16(35.6)	3(8.6)	4.779	<0.05
	<90 g/L	29(64.4)	32(91.4)		
WHO staging	RAEB-1	19(42.2)	27(77.1)	6.011	<0.05
	RAEB-2	26(57.8)	8(22.9)		
IPSS score	Low risk+medium risk	29(64.4)	20(57.1)	0.488	>0.05
	High risk	16(35.6)	15(42.9)		
Chromosome karyotype	Good+moderate	42(93.3)	24(68.6)	5.027	<0.05
	Bad	3(6.7)	11(31.4)		

## DISCUSSION

At present, clinical treatment schemes for MDS include blood transfusion and infusion of PLT, colony stimulating factor, biological response regulator, immunosuppressant, hematopoietic stem cell transplantation, etc.^9^,^10^ Although hematopoietic stem cell transplantation is the only promising method to cure at present, MDS patients who are mostly elderly and often have a variety of basic diseases cannot tolerate hematopoietic stem cell transplantation.^11^ Although the traditional chemotherapy scheme can achieve short-term remission effect, the effect lasts for a short time, and high-intensity treatment will cause chemotherapy-related complications, increase mortality and result in poor prognosis.^12^ It has been found that DNA abnormal methylation plays an important role in the occurrence and progress of MDS.^13^ DNA methylation is an important epigenetic modification. Abnormal DNA methylation can lead to the silencing of tumor suppressor genes and the abnormal regulation of normal cell growth and differentiation, and the damaged DNA cannot be repaired in time.^14^,^15^

DAC is a kind of adenosine analogue of deoxycytidine acid, which belongs to methyltransferase inhibitor. It can reduce DNA methylation by inhibiting DNA methyltransferase, make the tumor suppressor gene return to normal, reactivate the gene inactivated due to DNA excessive methylation, and make the process of cell differentiation, aging or apoptosis return to normal.^16^,^17^ A study of Ye et al. enrolled 81 cases of MDS-RAEB,^18^ 40 patients were treated with decitabine before chemotherapy, and the median follow-up time was 10.9 months. Through comparing the results of chemotherapy alone with that of DAC before chemotherapy, it was found that the objective response rate (ORR) and CR rate of the decitabine group was significantly higher than that of chemotherapy group. It further suggested that the effect of DAC in treating MDS was significant. In this study, the total effective rate of patients treated with DAC combined with CAG/DA was higher than that of patients treated only with CAG/DA, which was consistent with the previous reports. However, the ORR and CR rate of this study were lower than those of Jeong et al..^19^ The reasons are as follows. This study included more high-risk and extremely high-risk patients; thus the risk degree was higher. Moreover, the medium treatment course of patients with effective DAC treatment result was eight in the study of Jeong et al. and that of patients who received decitabine treatment in this study was four. Some patients stopped treatment because of economic reasons, compliance or severe complication in the process of medicine use, and DAC has not taken effect at that time, leading to reduced effective rate.

The analysis of the survival condition between the two groups suggested that the difference of OS between the two groups had no statistical significance and the combined group had significantly higher PFS than the traditional group, which suggested that decitabine could increase the disease-free survival time of MDS patients. Wen et al. found that the median OS of high-risk MDS patients who were treated by decitabine was significantly higher than that of chemotherapy.^20^ The difference between the result of Wen et al. and this study might be that the sample size was small and the follow up time was short.

In addition, this study also innovatively analyzed factors that affected the treatment effect of MDS patients. It was found that the Hb level, WHO stage and chromosome karyotype of patients before treatment had a significant impact on the treatment effect of MDS and the treatment effect of patients with Hb<90 g / L, RAEB-1 stage and bad chromosome karyotype was often poor.

## CONCLUSION

In conclusion, the effect of DAC combined with conventional chemotherapy in the treatment of MDS is good, which will not increase the adverse reactions of patients. In addition to the treatment scheme, the influence factors of effect of MDS also include Hb, WHO stage and chromosome karyotype of patients.

### Authors’ Contribution

**TXL:** Study design, data collection and analysis.

**JW & CML:** Manuscript preparation, drafting and revising.

**TXL & LZJ:** Review, final approval of manuscript and are responsible for integrity of study.
